# Development and Validation of a New GC-FID Method for the Determination of Short and Medium Chain Free Fatty Acids in Wine

**DOI:** 10.3390/molecules27238195

**Published:** 2022-11-24

**Authors:** Lucia Lenti, Ancuta Nartea, Oghenetega Lois Orhotohwo, Deborah Pacetti, Dennis Fiorini

**Affiliations:** 1 School of Science and Technology, Chemistry Division, University of Camerino, Via Madonna delle Carceri 9/B, 62032 Camerino, Italy; 2 Department of Agricultural, Food and Environmental Science, Polytechnic University of Marche, 60131 Ancona, Italy

**Keywords:** short chain fatty acids (SCFAs), medium chain fatty acids (MCFAs), wine, volatile compounds, flavor, gas chromatography

## Abstract

A new analytical method for the determination of six volatile short and medium-chain fatty acids (acetic, propionic, isobutyric, isovaleric, hexanoic, and octanoic acids) through liquid–liquid extraction with diethyl ether, followed by GC-FID analysis, was developed and validated. The extraction conditions were optimized by evaluating the effect of the number of extractions (1 to 3) and the effect of the addition of salts (NaH_2_PO_4_, (NH_4_)_2_SO_4_, NaCl, (NH_4_)_2_SO_4_/NaH_2_PO_4_) to increase the concentration of the analytes in the ethyl ether phase. Results showed that a single extraction allows obtaining the highest sensitivity (due to the impossibility of evaporating the solvent to avoid losses of the analytes). The use of salting out agents, in particular, NaH_2_PO_4_, showed an important increase in the extraction extent, on average, 1.5 times higher as compared to the extraction performed without salt. The proposed method is rapid, requiring a total of 30 min for preparation and analysis, and it makes use of small amounts of sample (500 µL) and solvent (400 µL). The method was then applied to quantify the analytes in 5 white wines and 5 red wines, allowing to highlight some clear differences between red and white wines, with the red ones having a significantly higher amount of acetic acid (715.7 ± 142.3 mg/L in red wines and 351.5 ± 21.2 mg/L in white wines) and the white wines having a significantly higher amount of hexanoic and octanoic acid (6.1 ± 3.0 mg/L and 2.6 ± 0.8 mg/L, respectively, are the mean concentrations in white wines, and 4.7 ± 0.8 and 2.4 ± 0.4 mg/L, respectively, are the mean concentrations in red wines).

## 1. Introduction

Short and medium-chain free fatty acids (SCFAs and MCFAs, including acetic to hexanoic acid and heptanoic to dodecanoic acid, respectively) are volatile organic acids having the structure of fatty acids, but with short carbon chains, providing them with higher polarity, volatility, and thus different properties than long-chain fatty acids, commonly constituting food lipids. SCFAs and MCFAs play important roles in foods and several biological samples. In foods, their variable amount and composition contribute to the characteristic flavor, thus being in relation with their quality [1]. In wine, acetic acid represents volatile acidity, being the SCFA largely predominant. An uncontrolled proliferation of acetic acid bacteria, leads to high levels of acetic acid, resulting in the typical vinegar-like defect in the aroma and the consequent wine spoilage [2]. While in the case of vinegar, acetic acid gives a fundamental contribution to the aroma and there is a minimum level that should be found in the product (0.5% *w/v* is the value established in European Union, Regulation n.238/2016 art. 49). The same molecule should not exceed a certain level in wine (according to Regulation (CE) n. 606/2009, 18 mEq/L for white and rosé wines, 20 mEq/L for red wines, corresponding to 1.08 g/L and 1.20 g/L respectively) to avoid sensorial defects.

Even if at much lower concentrations, other SCFAs and MCFAs are also found in wine. They are bio-synthetized during alcoholic fermentation by the action of yeasts and bacteria and they can have different origins. While SCFAs such as propionic, isobutyric, and isovaleric are by-products of protein metabolism, linear and longer saturated fatty acids with an even number of carbon atoms, i.e., hexanoic, octanoic, and decanoic acids, are produced from lipid metabolism, i.e., from the catabolism of long-chain fatty acids [3,4]. When these acids are present in concentration levels lower than their olfactory thresholds, they positively contribute to the final aroma with the so-called “complexity”. Conversely, when present in higher concentrations, they may negatively impact wine bouquets with off-flavors associated with buttery, rancid, and goaty scents [5]. At the same time, acids such as hexanoic, octanoic, and decanoic, are relevant for the formation of the relative esters, which can give pleasant and fruity/floral character. Some authors have found a positive correlation between their presence and the characteristic bouquet of young white wines [6].

The analysis of SCFAs can be performed in different matrices by exploiting different techniques, such as high-performance liquid chromatography (HPLC), capillary electrophoresis (CE), nuclear magnetic resonance (NMR), or gas chromatography (GC) [7,8,9,10]. However, due to the volatility of these analytes, GC is among the most used instrumental techniques for their analysis. Regarding the pre-treatment used to isolate the analytes of interest and to remove impurities, liquid–liquid extraction (LLE) has been reported to be a valid method due to the different polarities of SCFAs and MCFAs, permitting the use of different solvents or mixture of solvents, adapting its polarity to the analytes. The obtained liquid extract can be directly used for the quantitative analytical measurements in gas or liquid chromatography, without the need for a desorption step from the extractive phase needed in extraction techniques such as e.g., solid phase microextraction (SPME) or solid phase extraction (SPE) [11]. Several studies have been reported involving the use of SPME to extract volatile compounds, including also SCFAs and MCFAs from wine (e.g., [12,13]), or from other matrices (e.g., [10,14]).

Regarding approaches exploiting LLE, one of the first studies concerning the analysis of SCFAs and MCFAs (C6–C10) in wine, investigated samples from Japan and other countries, performing the extraction with a mixture of ethyl acetate-*n*-pentane (2:1, *v*/*v*), followed by GC analysis [15]. The quantification of C6–C18 acids and their related ethyl ester, by extracting the analytes through LLE with hexane, was performed in a study to evaluate the effect of the presence of fatty acids in bound or free form on sparkling wines foaming [16]. Liquid–liquid extraction, using a mixture of diethyl ether-hexane (1:1, *v*/*v*), was also used to investigate the volatile composition, including volatile acids, of two Mencía wines [17]. The extraction performed through LLE with hexane, followed by GCxGC-TOF-MS analysis, was also exploited for the analysis and quantification of the major volatile alcohols, esters, and acids in white Gewürztraminer wines treated with three different yeast strains [18]. Furthermore, derivatization was taken into consideration by some authors for the quantification of free fatty acids [19]. In particular, medium and long-chain free fatty acids (C6–C18) were determined by adding concentrated hexane extracts with an H_2_SO_4_ solution (3% in methanol) to obtain the relative methyl esters. A correction factor was necessary to discriminate between the methyl esters obtained from free fatty acids and those obtained from the partial transmethylation of ethyl esters normally present in wine.

Due to the importance of SCFAs and MCFAs as contributors defining wine quality, and to the limited number of reports on methods specifically designed for their quantitative GC analysis in wine, this present study aimed to optimize and validate a new analytical method for their direct analysis, avoiding any derivatization. To this purpose, LLE has been exploited by investigating the number of extractions and the impact of different salts as salting out agents, analyzing the extracted SCFAs and MCFAs by GC coupled with flame ionization detection (FID). 

## 2. Results and Discussion

### 2.1. Optimization of the Extraction Procedure: Number of Extractions

**Figure 1:**
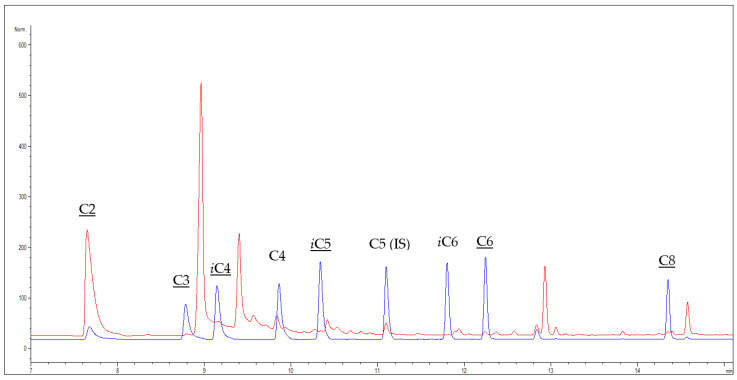
Overlapped chromatograms obtained from GC-FID analysis of a red wine sample (Nero D’Avola) containing the internal standard (IS) (red chromatogram) and a standard mixture containing SCFAs and MCFAs C2-C8 at 0.1 g L^−1^ (blue chromatogram). Underlined shorthand names indicate analytes that can be quantified by the present method.

In this study, analytes (SCFAs and MCFAs) are extracted at room temperature from wine by LLE using ethyl ether as an extraction solvent that has been reported to be a good solvent for extracting SCFAs and MCFAs from different matrices [20,21,22]. The analysis of SCFAs and MCFAs extracted is then performed by GC-FID with splitless injection, to obtain a suitable sensitivity. The chromatographic column used is a capillary column coated with polyethylene glycol but having high inertness (WAX-UI). This column was preferred over the nitro terephthalic acid modified polyethylene glycol (FFAP) (taking into consideration the same length, 30 m, same 0.25 µm coating thickness, and 0.25 mm i. d.) because the highly polar FFAP, even if specific for analyzing free fatty acids [20,23], gave coelution of propionic acid and 2,3-butandiol, while butyric acid gave coelution problems with both the columns. Analytes that could be identified and quantified using WAX-UI were acetic acid (C2), propionic acid (C3), isobutyric acid (*i*C4), isovaleric acid (*i*C5), hexanoic acid (C6), and octanoic acid (C8) (Figure 1). Their identity was also confirmed by performing the analysis in the same conditions by GC-MS. 

The number of extractions to be performed is an important parameter to be considered in LLE. Furthermore, if analytes show great differences from each other in terms of polarity, the ideal number of extractions could be different for the different analytes. In the case of SCFAs and MCFAs, their solubility properties change greatly moving from acetic acid, which is soluble in water, to hexanoic or octanoic acids, which are insoluble in water. Their partition between water and ethyl ether phases changes greatly moving from acetic acid to the longer chain homologous, decreasing significantly with the increase of carbon chain length, e.g., the difference in the partition between acetic acid and propionic acid, between water and ethyl ether phase, is much higher than the one between isovaleric and hexanoic acids, even if the carbon chain length difference is the same. This is due to the different impact of the polar carboxylic end in molecules having a low or a high number of carbons in the hydrophobic chain This is also clearly shown by the octanol-water partition coefficient (K_ow_), commonly used to assess the polarity (or apolarity) of a substance, which for this homologous series of analytes, has an exponential trend with the increasing number of carbons in the chain. The more commonly used logarithmic values (log *P*) are 0.17 for acetic acid, 0.33 for propionic acid, 0.79 for butyric acid, 1.39 for valeric acid, 1.92 for hexanoic acid, 3.05 for octanoic acid [24]. Thus, a single extraction with diethyl ether could be enough to extract less polar analytes, such as hexanoic or octanoic acids, while the most polar, i.e., acetic acid, would give a much lower extraction extent [20]. However, in the specific case of wine, acetic acid is largely the most abundant volatile acid, being present in concentrations hundreds of times greater than the other SCFAs and MCFAs [25]; thus, it is possible that even with only one or two extractions, a sufficient sensitivity could be obtained for the quantification (even if the extraction extent would not be complete with only one extraction). 

In principle, a higher number of extractions would ensure the maximum recovery for all the analytes, but in the specific case under investigation, the volatility of the analytes prevents the possibility of evaporating the solvent to concentrate the analytes. Thus, a higher number of extractions, even if guaranteeing a higher recovery of the analytes, would lead to a dilution with subsequent loss in sensitivity. For this reason, the number of extractions to obtain a maximum sensitivity for most of the analytes, was assessed by performing 1, 2, or 3 subsequent extractions and the results are reported in Table 1. 

A significant decrease in the chromatographic signals is obtained for each of the analytes increasing the number of extractions from 1 to 2. However, while the signal for acetic acid is high either with 1, 2, and 3 extractions, for the other analytes the signals get close to those corresponding to LOQ values when performing two subsequent extractions. Thus, it was decided to proceed with a single extraction, to maintain an adequate level of sensitivity for all the analytes. 

### 2.2. Optimization of the Extraction Prodecure: Effect of Different Salts

**Figure 2:**
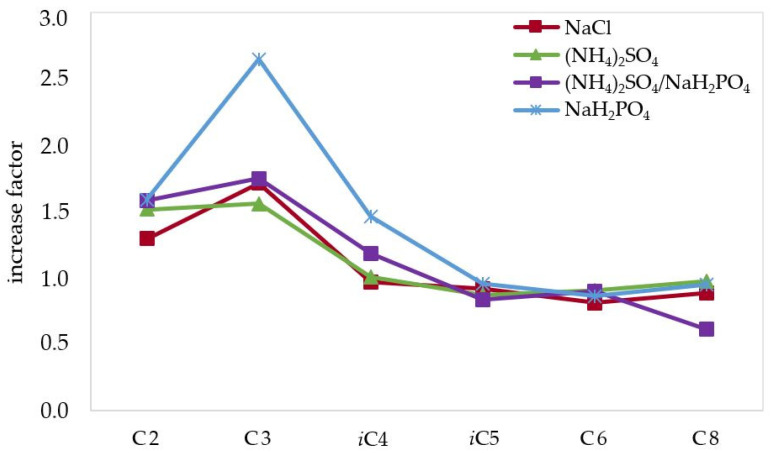
Increase factor calculated as the ratio between the mean peak area obtained from each SCFA and MCFA extracted from a wine sample (Nero D’Avola) using different salts and the mean peak area obtained without using salt.

To increase the extraction extent of the analytes of interest from the wine matrix, and thus sensitivity, the addition of different salts has been also investigated, since their effect on the ionic strength of the solution can greatly affect the partition of the analytes between the hydro-alcoholic and the organic phase [26]. Salts and salts mixtures having high solubility in water and/or producing multiple charged ions were selected: NaH_2_PO_4_, (NH_4_)_2_SO_4_, (NH_4_)_2_SO_4_/NaH_2_PO_4_ (3.7:1), and NaCl; and they were added to the sample obtaining saturated solutions. A previous study demonstrated the capability of these salts to improve, greatly in some cases, the headspace SPME of SCFAs and MCFAs from different food and biological samples [27]. The extraction extent obtained with the above-mentioned salts is shown in Table 2, with the increase factor compared to the extraction performed without salt (Figure 2), measured in terms of a ratio between the signal of the analyte performing the extraction by using salt or salt mixture and without using any salt.

The addition of salts resulted to produce the maximum increase in the case of acetic and propionic acid, which resulted to be extracted 2–2.5 times more when using NaH_2_PO_4_, the salt that overall provided the best performance (on average 1.5 for all the analytes), even if the improvement regarded only the lighter, more polar analytes. This can be explained considering that the lighter, and more importantly, more polar analytes have a much different partition between the aqueous and ethyl ether phases, as compared to their longer chain homologous, thus being more affected by changes in ionic strength of the aqueous phase. The mixture (NH_4_)_2_SO_4_/NaH_2_PO_4_ produced a maximum increase of 1.9 for acetic acid and the average for all the analytes was 1.2, while NaCl produced a maximum increase of 1.6 for propionic acid and an average increase of 1.1. Even if the result is not generalized for all the analytes, it was decided to use NaH_2_PO_4_, since it greatly improved the extraction of acetic, propionic, isobutyric, and octanoic acid in a significant way. The impact of salts hydrolysis (NaH_2_PO_4_, (NH_4_)_2_SO_4_, (NH_4_)_2_SO_4_/NaH_2_PO_4_) on wine pH was monitored and was negligible in all the cases, allowing to maintain SCFAs and MCFAs in their undissociated forms, thus extractable with the organic solvent used for the LLE (ethyl ether).

### 2.3. Method Validation

The linearity of the method was assessed by exploiting the method of standard additions to a wine model to reproduce, in the calibration, the matrix effect that otherwise could bias the quantification results. A good linearity was obtained in the range of concentrations of targeted analytes found in the samples, with R^2^ values between 0.994 and 0.997 (Table 3).

Limits of detection (LOD) values were in the range of 0.04–0.51 mg L^−1^, while limits of quantification (LOQ) were in the range of 0.13–1.70 mg L^−1^, with values low enough to permit the quantification of the analytes. The results are similar to those reported in a study where the analytes were extracted through HS-SPME and analyzed by GC coupled to an ion trap mass spectrometer, with LOD values in the range of 0.003–0.275 mg L^−1^ [28]. In another study performed by HS-SPME-GC-MS, LOQ values reported for the quantification of C6 and C8 in Semillon wines, are 8.34 and 0.40 mg L^−1^, respectively [29], higher, in particular for C6, as compared to the results obtained in the present study (0.18 mg L^−1^ for C6, 0.21 mg L^−1^ for C8). Instead, significantly lower values (0.03 mg L^−1^ for C6 and 0.02 mg L^−1^ for C8) have been reported in a work where free fatty acids were determined in musts and wines through LLE with hexane followed by concentration and derivatization of the analytes with 3% sulfuric acid in methanol and subsequent GC-FID analysis [19].

The recovery of the method was assessed by spiking the wine sample with an amount of each analyte equal to its average amount found in the samples. Recovery values obtained were in the range of 81.9–101%.

Repeatability, in terms of relative standard deviation obtained in the analysis of a wine sample performed 5 times in a day (intraday repeatability) and 5 times in 5 different days (interday repeatability), showed good values ranging from 0.4 to 4.9% for the intraday and from 0.5 to 8.5% for interday repeatability (Table 3). 

#### Application of the Method to Samples

The validated method was then applied to quantify C2, C3, *i*C4, *i*C5, C6, and C8 in different white and red wine samples (Table 4). The distribution of the analytes is different in red and white wines, especially for the content of acetic acid, hexanoic acid, and octanoic acid, with acetic acid being significantly more abundant in red wines and hexanoic and octanoic acid being significantly more abundant in white wines. The same trend is reported in several other studies [15,28,30]. As an example, in a study on the characterization of South African young red and white wines performed by LLE followed by GC-FID analysis [25], acetic acid concentration was higher in red wines (491.3–597.5 mg L^−1^) than in white ones (395.4–408.1 mg L^−1^), while hexanoic and octanoic acids showed lower concentrations in red wines (1.43–1.9 and 1.3–1.8 mg L^−1^ respectively), as compared to white wines (5.2–5.8 and 4.7–6.2 mg, respectively). Similarly, we found acetic acid concentration in white wines in the range 351.5–381.5 mg L^−1^ and in red wines 440.0–821.6 mg L^−1^. However, the red wine Lacrima di Morro d’Alba resulted to have an acetic acid content of 440.0 mg L^−1^, much lower as compared to the other red wines (718.7–821.6 mg L^−1^). Taking into consideration the limit imposed by European regulation (CE) n. 606/2009 for the content of acetic acid (1080 mg L^−1^ for white and rosé wines and 1200 mg L^−1^ for red wines) all the wines investigated resulted to have lower concentrations. Regarding C6 and C8, their mean amount found in white wines is 4.7 and 6.1 mg L^−1^ respectively, while in red wines they are found in average concentrations of 2.4 and 2.6 mg L^−1^, respectively. Moreover, in this case, Lacrima di Morro d’Alba is a sample having peculiar features as compared to the other red wines investigated, with the highest concentration of C6 and C8, intermediate between the other red wines and white wines investigated, as found in the case of acetic acid. Propionic acid, isobutyric acid, and isovaleric acid are found in slightly lower concentrations as compared to C6 and C8 and they are generally less abundant in white wines as compared to red wines. Moreover, an investigation on aroma components of Galician Albariño, Loureira, and Godello white wines, produced with different grape varieties, reports higher concentrations of hexanoic and octanoic acids as compared to the other volatile acids (different than acetic acid). 

Regarding the relative amounts of C6 and C8, we found that C8 is on average more abundant than C6, especially in white wines, even if the difference found is not statistically significant probably due to the very low level of C8 found in only table wine investigated. The trend found for C6 and C8 has been evidenced also in the study reported by Shinoara (1985) [15].

For each analyte, the OAV values were also calculated for all the samples under investigation, by dividing the concentration of a given compound by its olfactory threshold reported in the literature [31,32,33] (Table 4). In the case of propionic acid, the OAVs were always below the unit (ranging from 0.03 to 0.1) meaning that the compound is not relevant from the sensorial point of view. Acetic acid OAV values are in the range of 1.6–4.0, thus it may play an active role in the aroma. However, it never exceeds the limits dictated by regulations. Concerning the other compounds, except for isobutyric acid, high OAV values were obtained in all the samples. Isovaleric acid had an OAV in the range of 8.7–33.3 for white samples and 9.1–45.4 for red wines. Very high OAV values for isovaleric acid, namely 51.8 for white samples and 58.1 for red wines, were also reported in another study [30]. For hexanoic and octanoic acids, lower values were obtained, but still in this case higher than one, which is also in agreement with the mentioned study. 

## 3. Materials and Methods

### 3.1. Standards, Reagents, and Solvents 

The analytical standards acetic acid (C2, purity ≥ 99%), propionic acid (C3, purity ≥ 99,5%), *i*-butyric acid (*i*C4, purity ≥ 99%), *n*-butyric acid (C4, purity ≥ 99%), isovaleric acid (*i*C5, purity ≥ 98%), valeric acid (C5, purity ≥ 99%), isohexanoic acid (*i*C6, purity ≥ 99%), hexanoic acid (C6, purity ≥ 98%), octanoic acid (C8, purity ≥ 98%), NaH_2_PO_4_, and (NH_4_)_2_SO_4_ were purchased by Sigma Aldrich. Ethyl ether was purchased by J.T. Baked (Phillipsburg, NJ, USA), sulfuric acid, and NaCl were purchased from Carlo Erba (Milan, Italy). The water used was deionized (resistivity above 18 MΩ cm). 

Standard stock solutions were prepared at different concentrations by dissolving pure analytical standards (200 µL for C2 and 10 µL for the other analytes, C3–C8) in diethyl ether until reaching 10 mL in volumetric flasks. The mother stock solutions were then used to prepare mix standard solutions, by performing proper dilutions.

### 3.2. Wine Model

A wine model was prepared to build calibration curves with the standard additions method and to perform recovery tests. The model was a hydroalcoholic solution based on the macroscopic composition of a wine sample, prepared according to previous work [34]. Briefly, the wine model was prepared by dissolving absolute ethanol (14.16 mL), glycerol (1 g), D-tartaric acid (0.53 g), D-glucose (0.26 g), D-fructose (0.26 g) in deionized water to a final volume of 100 mL, in a volumetric flask.

### 3.3. Samples

The samples used for the optimization and application of the method were purchased in supermarkets or provided by wineries in the Marche region (Italy) and they were 5 white wines (“Passerina 1” from “Terre di Chieti”, “Passerina 2” and “Pecorino 1” from “Terre Cortesi Moncaro”, “Pecorino 2” from “Cantine di Castignano” and table wine) and 5 red wines (“Lacrima di Morro d’Alba” from “Vicari”, “Nero d’Avola” from “Ciacaranni”, that was used for the method optimization, “Offida Rosso 1” from “Cantine Dei Colli Ripani”, “Offida Rosso 2” from “Velenosi”). 

### 3.4. Sample Preparation

In a 2 mL vial, 0.5 mL of wine sample and 0.6 g of salt NaH_2_PO_4_ (to obtain a saturated solution) are added and vigorously mixed with a vortex device for 1 min. The sample is added with 25 µL of internal standard solution (170 µL L^−1^ C5 ethyl ether solution) and then 0.4 mL of diethyl ether is added for the extraction. The sample is stirred again using a vortex device for 3 min and then the two layers are separated with the help of a centrifuge (5000 rpm, 5 min). The upper organic phase is collected and analyzed by GC-FID. The whole extraction procedure is performed at room temperature.

### 3.5. Analysis of SCFAs and MCFAs by Gas Chromatography Coupled to Flame Ionization Detection

The gas chromatography analysis was performed using an Agilent Technologies 6850 GC (Santa Clara, CA, USA) equipped with a split/splitless injector and coupled with a flame ionization detector (FID). The injection (1 µL) was performed in splitless mode (splitless time 3 min) with the injector temperature set at 280 °C. The carrier gas was hydrogen produced by a generator (MARS 250 N from ErreDue, Livorno, Italy) and the initial hydrogen flow in the column was 2.50 mL/min. The capillary chromatographic column was an ultra-inert polyethylene glycol column (DB-WAX UI), length 30 m, 0.25 mm i. d., 0.25 µm film thickness, purchased from Agilent Technologies (Santa Clara, CA, USA). The oven temperature was set at 40 °C and maintained for 3 min, then raised at 10 °C/min to 210 °C and then at 40 °C/min to 245 °C and maintained for 1.87 min, for a final run time of about 20 min. The FID temperature was set at 250 °C. The identification of SCFAs and MCFAs in real wine samples was performed by comparison of their retention times with reference standard solution. Their identity was also confirmed by performing the analysis of a standard mixture and a wine sample by GC-MS using a 6890 N GC coupled with a 5973 N single quadrupole mass spectrometer detector (Agilent Technologies, Santa Clara, CA, USA). 

### 3.6. Quantification and Method Validation

The validation of the method was performed by assessing linearity, repeatability, recovery, and limits of detection (LOD) and quantification (LOQ). The linearity of the method was assessed by calculating the linear regression coefficient (R^2^) obtained from the calibration curves which were constructed with the method of standard additions in a wine model. The repeatability was assessed by analyzing and preparing five replicates of a wine sample within the same day and five replicates prepared on different days and calculating the % relative standard deviation obtained for each analyte. LOD and LOQ were defined by considering the peak areas corresponding to 3 and 10 times the signal to noise ratios, respectively. 

The recovery of the method was assessed by spiking the wine sample with 10 µL of a standard mixture solution containing all the analytes at different concentrations (C2 at 13,000 mg L^−1^, C3 at 30 mg L^−1^, *i*C4 at 50 mg L^−1^, *i*C5 at 20 mg L^−1^, C6 at 90 mg L^−1^ and C8 at 100 mg L^−1^) and for each analyte, the average amount found in the samples was investigated.

The sample was then extracted following the procedure reported in Section 3.4 and then analyzed by GC-FID. Recovery was calculated for each analyte by dividing the difference between the signal obtained analyzing the spiked sample and the sample as such, by the signal of the added standard analyte. 

### 3.7. Statistical Analysis

One-way analysis of variance (ANOVA) followed by Tukey’s test for pairwise comparison was performed to assess significant differences (*p* < 0.05). The software used for this purpose was PAST [35].

## 4. Conclusions

A new method for the determination of short and medium-chain fatty acids in wine was developed and validated, being these important molecules able to impact the aroma, and thus the overall wine quality. The proposed method allows performing the analysis in a short time (30 min), with a simple pre-treatment of the sample, avoiding the derivatization of the analytes. Small amounts of sample are used (0.5 mL), few inexpensive reagents, and an analytical instrument commonly available in most laboratories (GC-FID). The use of salts able to improve the extraction extent was evaluated and NaH_2_PO_4_ allowed to significantly increase the method sensitivity. Good linearity, precision, repeatability, and sensitivity make this procedure suitable for the determination of acetic, propionic, isobutyric, isovaleric, hexanoic, and octanoic acids in wine, thus providing a useful tool to investigate its quality. 

## Figures and Tables

**Table 1:** Mean concentration (mg L^−1^) ± standard deviation (*n* = 2) obtained for each analyte from GC-FID analysis performing 1,2 or 3 subsequent ethyl ether extractions from a red wine sample (Nero D’Avola).

Compound	1st extr	2nd extr	3rd extr
acetic acid	493.7 ± 5.4 ^a^	429.8 ± 3.6 ^b^	400.4 ± 3.4 ^c^
propionic acid	0.8 ± 0.0 ^a^	0.6 ± 0.0 ^b^	0.4 ± 0.0 ^c^
isobutyric acid	2.5 ± 0.3 ^a^	1.5 ± 0.0 ^b^	1.3 ± 0.1 ^b^
isovaleric acid	1.2 ± 0.0 ^a^	1.1 ± 0.1 ^b^	0.9 ± 0.0 ^c^
hexanoic acid	2.4 ± 0.1 ^a^	1.5 ± 0.1 ^b^	1.1 ± 0.1 ^c^
octanoic acid	3.3 ± 0.0 ^a^	2.0 ± 0.2 ^b^	1.3 ± 0.1 ^c^

Letters indicate significant differences between the signals (in terms of peak area) obtained performing from 1 to 3 subsequent extractions (extr) (One-way ANOVA, *p* < 0.05, Tukey’s test for pairwise comparison).

**Table 2:** Extraction extent of SCFAs and MCFAs by liquid-liquid extraction with ethyl ether from wine (Nero d’Avola) using different salts, salts combination or in absence of salt. Results are reported in terms of concentration (mg L^−1^) ± standard deviation (*n* = 2) from GC-FID analysis.

Compound	No Salt	NaCl	(NH_4_)_2_SO_4_	(NH_4_)_2_SO_4_/ NaH_2_PO_4_	NaH_2_PO_4_
acetic acid	490.2 ± 2.6 ^a^	633.3 ± 3.7 ^a^	742.0 ± 10.5 ^b^	775.7 ± 1.3 ^b^	777.7 ± 5.8 ^b^
propionic acid	0.7 ± 0.1 ^a^	1.3 ± 0.1 ^a^	1.2 ± 0.1 ^a^	1.3 ± 0.2 ^a^	2.0 ± 0.3 ^b^
isobutyric acid	2.4 ± 0.0 ^a^	2.3 ± 0.0 ^a^	2.4 ± 0.3 ^a^	2.8 ± 0.1 ^a,b^	3.5 ± 0.3 ^b^
isovaleric acid	1.3 ± 0.1 ^a^	1.2 ± 0.0 ^a,b^	1.1 ± 0.0 ^b^	1.1 ± 0.0 ^b^	1.2 ± 0.0 ^a,b^
hexanoic acid	2.5 ± 0.2 ^a^	2.0 ± 0.2 ^a^	2.3 ± 0.0 ^a^	2.3 ± 0.1 ^a^	2.2 ± 0.1 ^a^
octanoic acid	2.9 ± 0.1 ^a^	2.5 ± 0.2 ^a^	2.8 ± 0.2 ^a^	1.8 ± 0.2 ^b^	2.7 ± 0.1 ^a^

Letters indicate significant differences between the extraction extent for each analyte using different salts (One-way ANOVA, *p* < 0.05, Tukey’s test for pairwise comparison).

**Table 3:** Validation parameters.

Compound	Linearity Range (mg L^−1^)	R^2^	LOD	LOQ	Recovery (%)	Repeatability (%) (*n* = 5)	
			(mg L^−1^)			Intraday	Interday
acetic acid	262.5–3024	0.997	0.51	1.70	96.8	2.5	8.5
propionic acid	0.5–5.9	0.995	0.04	0.14	86.4	0.4	2.1
isobutyric acid	0.5–4.3	0.999	0.06	0.19	96.6	1.6	1.6
isovaleric acid	0.5–4.1	0.996	0.04	0.13	81.9	0.0	1.1
hexanoic acid	0.6–7.4	0.997	0.05	0.18	101	2.9	0.5
octanoic acid	1.4–14.6	0.994	0.06	0.21	96	4.9	2.3

**Table 4:** Mean concentrations (mg L^−1^) ± standard deviations (*n* = 2) of SCFAs and MCFAs in wine samples, ranges of concentrations, mean values in red and white wines and calculated odor active values (OAV).

		C2	OAV	C3	OAV	*i*C4	OAV	*i*C5	OAV	C6	OAV	C8	OAV
white wines	Table wine	320.1 ± 4.7	1.6	1.1 ± 0.1	0.06	1.5 ± 0.0	0.6	0.3 ± 0.0	9.1	4.6 ± 0.4	10.9	1.8 ± 0.0	3.6
	Passerina 1	347.6 ± 4.9	1.7	0.9 ± 0.1	0.04	1.2 ± 0.1	0.6	0.3 ± 0.0	8.7	5.6 ± 0.3	13.1	6.3 ± 0.1	12.6
	Passerina 2	341.9 ± 1.4	1.7	0.6 ± 0.1	0.03	1.5 ± 0.1	0.6	1.0 ± 0.0	30.3	5.6 ± 0.1	13.2	9.8 ± 0.3	19.6
	Pecorino 1	380.3 ± 8.5	1.9	0.8 ± 0.0	0.04	1.7 ± 0.0	0.7	1.1 ± 0.0	33.3	3.2 ± 0.1	7.8	4.2 ± 0.6	8.4
	Pecorino 2	367.4 ± 4.9	1.8	1.0 ± 0.0	0.05	1.3 ± 0.0	0.6	1.0 ± 0.0	30.0	4.5 ± 0.3	11.7	8.3 ± 0.1	16.6
	Range	320.1–381.5		0.7–1.1		1.4–1.8		0.3–1.1		3.3–5.6		1.8–10.3	
	Mean values	351.5 ± 21.2 ^a^		0.9 ± 0.1 ^a^		1.5 ± 0.2 ^a^		0.7 ± 0.4 ^a^		4.7 ± 0.8 ^a^		6.1 ± 3.0 ^a^	
red wines	Lacrima Morro d’Alba	440.0 ± 1.5	2.2	1.8 ± 0.3	0.1	1.9 ± 0.0	0.8	0.4 ± 0.0	12.2	3.0 ± 0.4	7.1	3.8 ± 0.1	7.6
	Nero d’Avola	718.7 ± 1.2	3.6	1.6 ± 0.1	0.1	2.4 ± 0.2	1.0	0.3 ± 0.0	9.1	2.4 ± 0.1	5.7	3.2 ± 0.3	6.4
	Offida Rosso 1	802.5 ± 12.4	4.0	2.4 ± 0.2	0.1	3.0 ± 0.0	1.3	1.3 ± 0.0	39.4	1.9 ± 0.0	4.5	1.7 ± 0.0	3.4
	Offida Rosso 2	822.1 ± 7.8	4.1	1.0 ± 0.0	0.1	3.1 ± 0.0	1.3	1.5 ± 0.0	45.4	2.2 ± 0.0	5.2	2.2 ± 0.0	4.4
	Conero	795.4 ± 6.0	3.9	0.6 ± 0.0	0.0	2.8 ± 0.2	1.2	1.5 ± 0.0	45.4	2.5 ± 0.3	5.9	2.2 ± 0.0	4.4
	Range	440.0–821.6		0.7–2.5		1.9–3.1		0.3–1.6		1.8–3.0		1.6–3.8	
	Mean values	715.7 ± 142.3 ^b^		1.5 ± 0.7 ^b^		2.6 ± 0.5 ^b^		1.0 ± 0.5 ^a^		2.4 ± 0.4 ^b^		2.6 ± 0.8 ^b^	

Odor threshold (OTH) values used to calculate OAVs (OAV = concentration of the analyte (mg/L)/OTH) are 200, 20, 2.3, 0.033, 0.42 and 0.50 mg L^−1^ for C2, C3, *i*C4, *i*C5, C6, C8, [31,32,33] respectively. Letters indicate significant differences between concentrations of analytes in white and red wine samples (One-way ANOVA, *p* < 0.05, Tukey’s test for pairwise comparison).

## Sample Availability

Not available.
